# LncRNA SNHG6 promotes chemoresistance through ULK1-induced autophagy by sponging miR-26a-5p in colorectal cancer cells

**DOI:** 10.1186/s12935-019-0951-6

**Published:** 2019-09-09

**Authors:** Xinke Wang, Zhixian Lan, Juan He, Qiuhua Lai, Xiang Yao, Qingyuan Li, Yongfeng Liu, Huasheng Lai, Chuncai Gu, Qun Yan, Yuxin Fang, Yue Zhang, Aimin Li, Side Liu

**Affiliations:** Guangdong Provincial Key Laboratory of Gastroenterology, Department of Gastroenterology, Nanfang Hospital, Southern Medical University, No. 1838, Guangzhou Avenue North, Guangzhou, People’s Republic of China

**Keywords:** Colorectal cancer, SNHG6, ULK1-induced autophagy, Chemoresistance, ceRNA

## Abstract

**Background:**

Chemotherapy resistance is one of the main causes of recurrence in colorectal cancer (CRC) patients and leads to poor prognosis. Long noncoding RNAs (lncRNAs) have been reported to regulate chemoresistance. We aimed to determine the role of the lncRNA small nucleolar RNA host gene 6 (SNHG6) in CRC cell chemoresistance.

**Methods:**

Cell drug sensitivity tests and flow cytometry were performed to analyze CRC cell chemoresistance. Animal models were used to determine chemoresistance in vivo, and micro RNA (miRNA) binding sites were detected by dual-luciferase reporter assays. Bioinformatics analysis was performed to predict miRNAs binding to SNHG6 and target genes of miR-26a-5p. SNHG6/miR-26a-5p/ULK1 axis and autophagy-related proteins were detected by qRT-PCR and western blotting. Furthermore, immunofluorescence was employed to confirm the presence of autophagosomes.

**Results:**

SNHG6 enhanced CRC cell resistance to 5-fluorouracil (5-FU), promoted autophagy, inhibited 5-FU-induced apoptosis, and increased 5-FU resistance in vivo. Bioinformatics analysis showed that miR-26a-5p might bind to SNHG6 and target ULK1, and dual-luciferase reporter assays confirmed this activity. qRT-PCR and western blotting showed that SNHG6 was able to negatively regulate miR-26a-5p but correlated positively with ULK1.

**Conclusion:**

SNHG6 may promote chemoresistance through ULK1-induced autophagy by sponging miR-26a-5p in CRC cells.

## Background

Recent studies have shown that colorectal cancer (CRC) is the third most common cancer and fourth leading cause of cancer-related death; moreover, the CRC incidence increased by 34% from 1.3 million to 1.7 million between 2006 and 2016 [1, 2]. As colon cancer is generally not responsive to novel immune checkpoint therapies, combined chemotherapy remains among the primary therapy methods for advanced CRC [3]. In general, active cytotoxic drugs, including 5-fluorouracil (5-FU), inhibit the enzymatic activity of thymidylate synthase during DNA replication [4]. Although advanced CRC is mostly initially responsive to combined chemotherapy, some patients experience tumor recurrence due to drug resistance, and the 5-year survival rate is lower than 10% in these patients [5]. Therefore, it is essential to achieve a better understanding of the mechanism of chemotherapy resistance in CRC. Overall, cancer chemoresistance occurs due to a complex interplay between gene regulation and the environment [6].

Autophagy, an evolutionarily ancient and highly conserved catabolic process involves cellular self-digestion via a double-membrane organelle called an autophagosome [7–9]. Autophagy consists of a sequence of molecular events that lead to formation of a autophagosome, which engulfs intracellular material and eventually fuses with the lysosome for degradation of its contents [5, 8]. Autophagy has been reported to promote tumor progression and resistance to treatment, and human cancer cells implanted in immunodeficient hosts were found to be more sensitive to chemotherapy in the presence of pharmacological inhibitors of autophagy [10, 11]. Initiation of autophagy begins with activation of the ULK1 complex (the Atg1 complex in yeast), linking the cellular nutrient status to downstream events in autophagy [9, 12, 13]. The ULK complex is composed of ULK1 as well as three other members, mATG13, focal adhesion kinase family interacting protein of 200 kDa (FIP200) and ATG101, which initiate autophagosome formation [14, 15]. Autophagy has been reported to be a prime target of regulatory pathways.

Recent studies have revealed that long noncoding RNAs (lncRNAs) drive many important cancer phenotypes through their interactions with other cellular macromolecules, including DNA, RNA and protein [16]. One hypothesis for the functional mechanisms of lncRNAs is the competitive endogenous RNA (ceRNA) hypothesis [17] which posits that specific RNAs can impair microRNA (miRNA) activity through sequestration, thereby upregulating miRNA target gene expression [17]. In our previous study, we found that the lncRNA small nucleolar RNA host gene 6 (SNHG6) was significantly upregulated in CRC and could promote CRC cell proliferation, invasion and migration [18]. It has already been reported that many lncRNAs regulate tumor chemoresistance through ceRNA mechanisms; for example, lncRNA MALAT1 modulates chemoresistance in gastric cancer by sponging miR-22b-3p, and lncRNA H19 confers 5-FU resistance in CRC by sponging miR-194-5p [6, 19]. In this study, we found that SNHG6 is able to promote CRC chemoresistance and enhance autophagy through regulation of ULK1 by sponging miR-26a-5p, which has been confirmed to be regulated by SNHG6 to suppress osteosarcoma [20].

## Materials and methods

### Clinical specimens, cell lines and ethics statement

The clinical CRC specimens and paired normal tissues collected from 31 patients and used in this study were described previously [18]. This study was approved by the Ethics Committee of Nanfang Hospital of Southern Medical University (IRB approval no.: NFEC-2013-098, approval date: 18th December 2013), and written informed consent was obtained from each patient. The RKO and HT29 SNHG6 shRNA-knockdown cell lines and SNHG6 overexpressing RKO and HCT116 cell lines were described previously in detail [18]. 5-FU-resistant RKO cells (RKO/5-FU) were established by continuous culture in medium containing stepwise increasing concentrations of 5-FU in the range of 0.5–10 μM over a period of 8 months.

### MicroRNA transfection

MiR-26a-5p negative controls, mimics and inhibitors were purchased from Ruibiotech, China. The sequences used were as follows:

microRNA negative control (sense): 5′-UUCUCCGAACGUGUCACGUTT-3′; negative control (antisense): 5′-ACGUGACACGUUCGGAGAATT-3′; inhibitor negative control: 5′-CAGUACUUUUGUGUAGUACA-3′; has-miR-26a-5p double strand mimics: UUCAAGUAAUCCAGGAUAGGCU/CCUAUCCUGGAUUACUUGAAUU; has-miR-26a-5p inhibitor: AGCCUAUCCUGGAUUACUUGAA.

Lipofectamin™ 3000 (Invitrogen, America) was used according to the manufacturer’s instructions.

### RNA isolation, cDNA synthesis, and quantitative real-time PCR

Total RNA extraction, reverse transcription, and quantitative real-time polymerase chain reaction (qRT-PCT) were performed as described previously [18]. The sequences of the primers used were as follows:

ULK1 mRNA (sense): 5′-CAGCAAAGGCATCATCCAC-3′,

ULK1 mRNA (antisense): 5′-GGTTGCGTTGCAGTAGGG-3′,

GAPDH (sense): 5′-GATATTGTTGCCATCAATGAC-3′, and

GAPDH (antisense): 5′-TTGATTTTGGAGGGATCTCG-3′.

### Western blot analysis

Western blotting was performed as described previously [18]. Primary antibodies [anti-GAPDH, 1:5000, Proteintech, China; anti-LC3-I, -LC3-II, -p-ULK1, -ATG13, -ULK1, 1:1000, Cell Signaling Technology, America] were used according to the manufacturer’s instructions. Image J software was employed to analyze relative protein expression.

### Dual-luciferase reporter assay

Luciferase activity was measured using a Dual Luciferase Assay Kit (Promega, America) according to the manufacturer’s instructions. To assess the SNHG6/miR-26a-5p binding specificity, the human SNHG6 3ʹ-UTR sequence that interacts with the miR-26a-5p seed sequence was mutated (binding site from 5ʹ-TTACTTGA-3ʹ to 5ʹ-ACGTAACG-3ʹ, Obio Technology, China); to examine the ULK1/miR-26a-5p binding specificity, the human ULK1 3ʹ-UTR sequence that interacts with the miR-26a-5p seed sequence was mutated (Mut-1, the first putative binding site from 5ʹ-TACTTGAA-3ʹ to 5ʹ-CGTAACGT-3ʹ; Mut-2, the second putative binding site from 5ʹ-TACTTGAA-3ʹ to 5ʹ-CGTAACGT-3ʹ, Allabio Technology, China). The 3ʹ-UTRs of human SNHG6 and ULK1 containing putative binding sites were cloned into the psiCHECK-REPORT vector containing both Renilla and firefly luciferase reporters. 293T cells were cotransfected with the SNHG6 dual-luciferase reporter plasmid and miR-26a-5p mimics or negative control as well as the ULK1 dual-luciferase reporter plasmid and miR-26a-5p mimics or negative control.

### Cell sensitivity of RKO cells to chemotherapy and flow cytometry analysis

To analyze CRC cell sensitivity to chemotherapy, SNHG6-knockdown, -overexpressing and control CRC cells were treated with 5-FU (range 0–100/200 μM), and cell viability was assessed by the CCK-8 assay (Dojindo, Japan). The IC50 was calculated. Apoptosis was measured using a PE Annexin V Apoptosis Detection Kit (BD Biosciences, China) according to the manufacturer’s instructions, and cells were analyzed with a FACSCalibur flow cytometer (BD Biosciences, China). Data were evaluated using FlowJo software (Tree Star, Inc., Ashland, OR).

### Immunofluorescence staining

To evaluate autophagy, we used immunofluorescence staining and electron microscopy. mRFP-GFP-LC3 autophagy double-labeled virus (Hanbio, China) was transfected into RKO-shSNHG6 cells and RKO control cells according to the manufacturer’s instructions. DAPI was used to stain nuclei after 24 h. As our stable RKO-shSNHG6 cells already exhibited GFP fluorescence after shRNA transfection, we only used RFP fluorescence to evaluate LC3-II. Images were recorded by Olympus FV1200 confocal microscopy (Olympus, Japan).

### Animal model

Male athymic 4-week-old BALB/c nude mice were purchased from the Central Laboratory of Animal Science, Nanfang Medical University and maintained in a specific pathogen-free facility. All RKO-shSNHG6 and RKO-Scramble cell injections were described previously, and each group was divided into two smaller groups [18]. Ten days later, each smaller group of mice received PBS (500 μl) or PBS containing 5-FU (50 mg/kg) by intraperitoneal injection every 2 days. The mice were killed 16 days later, and the tumors were harvested. The tumor volume (V) was obtained by measuring the length (L) and width (W) of the tumor with vernier calipers, which was calculated using the formula V = (L × W^2^) × 0.5. The tumor growth inhibition ratio was calculated using the formula (1 − therapy group tumor volume/control group tumor volume) * 100%.

### Bioinformatics analysis

To predict SNHG6-binding microRNAs, we used the DIANA tools LncBase Predicted v.2 (http://carolina.imis.athena-innovation.gr/diana_tools) and LncACTdb 2.0 (http://www.bio-bigdata.net) and searched by location, resulting in many microRNAs that might bind to SNHG6. To search for target genes of miR-26a-5p, we used data from Targetscan, microrna, mirDB, and StarBase to generate an intersection in a Venn diagram, resulting in 144 possible target genes. Next, we used DAVID (https://david.ncifcrf.gov/) for KEGG analysis, which revealed the mTOR pathway, of which ULK1 is a target gene.

### Statistical analysis

SPSS 22.0 statistical analysis software was employed for statistical analysis of the experimental data. The significance of differences between groups was estimated by Student’s *t* test. Additionally, multiple group comparisons were analyzed with one-way ANOVA. Statistically significant correlations between SNHG6 and ULK1 expression levels in CRC tissues and cell lines were analyzed by Pearson’s correlation analysis. *P < 0.05, **P < 0.01, ***P < 0.001 and ****P < 0.0001 were considered significant; ns indicates no significance.

## Results

### SNHG6 enhances 5-FU resistance and reduces 5-FU-induced apoptosis in CRC cells

**Fig. 1:**
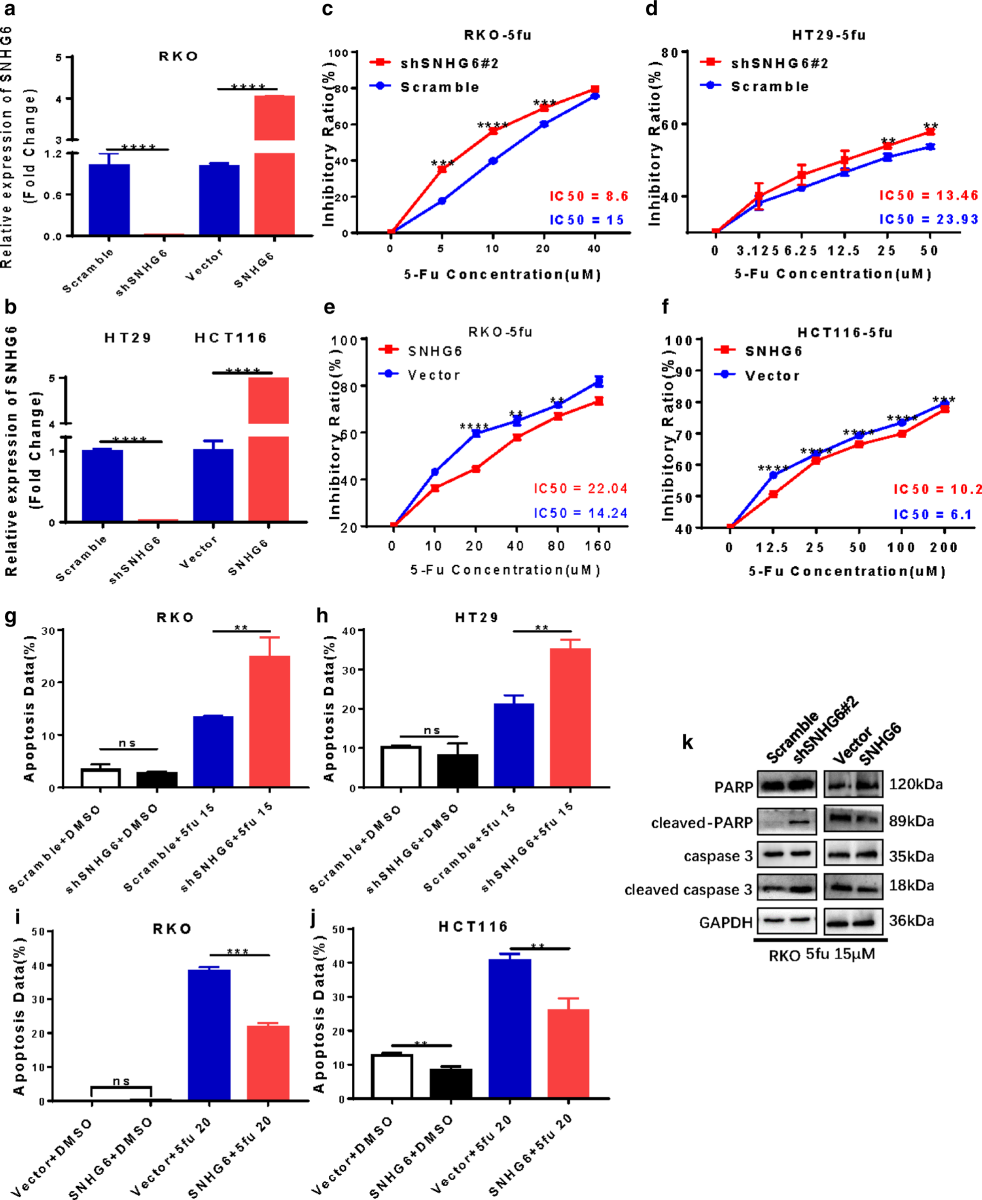
SNHG6 enhances drug-resistance to 5-FU and reduces 5FU-induced cell apoptosis in CRC cells. **a**, **b** SNHG6 knockdown and overexpression in CRC cells. **c**, **d** SNHG6 knockdown CRC cells were more sensitive to 5-FU, with lower IC50. **e**, **f** SNHG6 overexpression CRC cells were more resistant to 5-FU, with higher IC50. **g**, **h** SNHG6 knockdown CRC cells increased 5FU-induced apoptosis. **i**, **j** SNHG6 overexpression CRC cells reduced 5FU-induced apoptosis. **k** Western blot analysis of apoptosis well-defined proteins showed that SNHG6 could reduce RKO cells apoptosis. ns P > 0.05, *P < 0.05, **P < 0.01, *** P < 0.001, ****P < 0.0001, data was shown as the mean ± SD

We established SNHG6-knockdown RKO and HT29 cells transfected with SNHG6-specific shRNAs (Fig. 1a) and SNHG6-overexpressing RKO and HCT116 cells transfected with a plasmid harboring SNHG6 (Fig. 1b). 5-FU has been widely used for clinical chemotherapy in patients with CRC. In this study, we established 5-FU-resistant RKO cells (RKO/5-FU) and found that RKO/5-FU cells had lower levels of apoptosis and higher levels of autophagy than RKO cells did as well as higher half-maximal inhibitory concentrations (IC50), indicating that 5-FU can induce autophagy in RKO cells (Additional file 1: Figure S1a–c). We then used CRC cells with knockdown or overexpression of SNHG6 to evaluate its function in CRC cell drug resistance. We found that RKO and HT29 cells with SNHG6 knockdown became more sensitive to 5-FU, with lower IC50 values (Fig. 1c, d), but observed the opposite in RKO and HCT116 cells overexpressing SNHG6 (Fig. 1e, f).

We also employed flow cytometry to show that 5-FU induced CRC cell apoptosis and that SNHG6 knockdown enhanced drug-induced apoptosis in RKO and HT29 cells (Fig. 1g, h, Additional file 1: Figure S1d) but overexpression decreased it in RKO and HCT116 cells (Fig. 1i, j, Additional file 1: Figure S1d). Moreover, SNHG6 knockdown increased levels of well-defined apoptosis proteins, such as cleaved PARP and cleaved caspase-3, whereas overexpression of SNHG6 decreased these levels (Fig. 1k).

### SNHG6 promotes CRC cell autophagy and 5-FU resistance in vivo

**Fig. 2:**
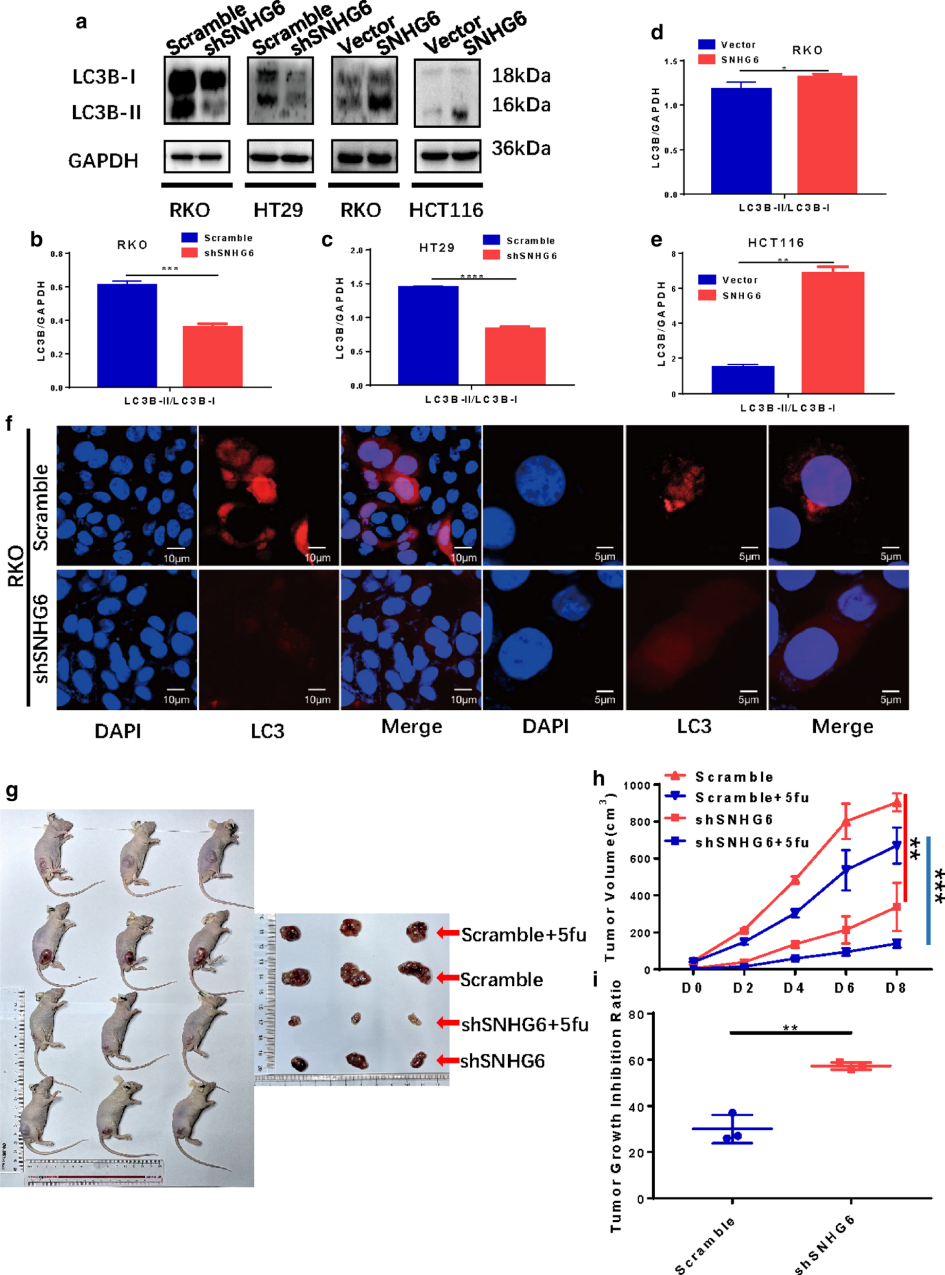
SNHG6 enhances autophagy in CRC cells and 5FU-resistance in vivo. **a** Western blot analysis showed SNHG6 could increase level of LC3-II, an autophagy-related protein, in CRC cell. **b**–**e** Relative expression of LC3B proteins. **f** Immunofluorescence RKO-shSNHG6 cells had fewer numbers of autophagosome. **g**, **h** RKO-shSNHG6 cells reduced tumor growth in vivo and promoted tumor sensitivity to 5-FU. **i** RKO-shSNHG6 cells had a higher tumor growth inhibition ratio with 5-FU treatment than control cells did. ns P > 0.05, *P < 0.05, **P < 0.01, ***P < 0.001, ****P < 0.0001, data was shown as the mean ± SD

We used western blot analysis to demonstrate that knockdown and overexpression of SNHG6 resulted in a lower level and higher level of LC3-II, respectively, an autophagy-related protein (Fig. 2a–e). Immunofluorescence staining also revealed that SNHG6 knockdown led to fewer autophagosomes (Fig. 2f), which indicates that SNHG6 induces autophagy in CRC cells.

Next, we used a mouse tumor model to evaluate whether SNHG6 promotes 5-FU resistance to increase the growth of tumors in vivo. We injected RKO cells stably transfected with shSNHG6 RNA and scramble RNA into the right hips of male nude mice and separated the mice into 2 groups, with 2 smaller groups for each. After 10 days, the mice in each smaller group were injected with 5-FU and PBS intraperitoneally every 2 days. According to the results, SNHG6 knockdown inhibited tumor growth compared with control cells, as proven previously [18]. Furthermore, SNHG6 knockdown improved 5-FU therapy and resulted in a higher tumor growth inhibition rate (Fig. 2g–j), which indicates that SNHG6 might enhance 5-FU resistance in CRC cells in vivo.

### SNHG6 regulates ULK1 by sponging miR-26a-5p in CRC tissues

**Fig. 3:**
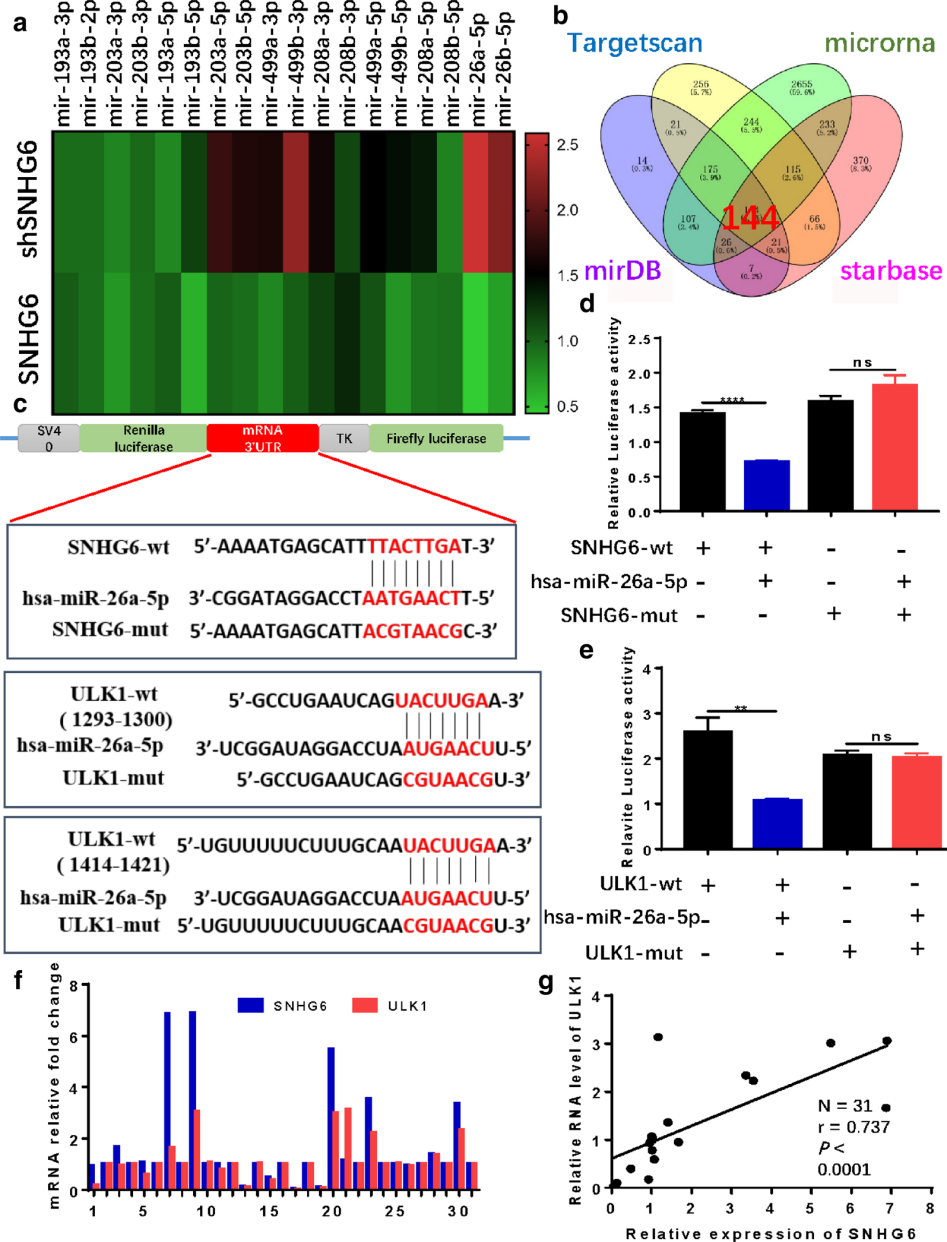
SNHG6 might combine with miR-26a-5p and regulate downstream gene ULK1. **a** qRT-PCR showed that miR-26a-5p was upregulated in RKO-shSNHG6 cells and downregulated in RKO-SNHG6 cells. **b** Venn Diagram of 144 miR-26a-5p downstream possible target genes. **c**–**e** Dual-luciferase reporter assays showed that miR-26a-5p bound to SNHG6 as well as ULK1. **f**, **g** qRT-PCR showed that SNHG6 had a positive correlation with ULK1 in 31 pairs of CRC tissues. ns P > 0.05, *P < 0.05, **P < 0.01, *** P < 0.001, ****P < 0.0001, data was shown as the mean ± SD

To explore the mechanism by which SNHG6 enhances autophagy and expression of downstream target genes, we used DIANA tools LncBase Predicted v.2 and LncACTdb 2.0 for bioinformatics analysis and found many microRNAs that may directly bind to SNHG6 [21]. qRT-PCR showed that miR-26a-5p was upregulated when SNHG6 was knocked down and downregulated when SNHG6 was overexpressed in RKO cells (Fig. 3a), and dual-luciferase reporter assays demonstrated that miR-26a-5p was able to bind to SNHG6 (Fig. 3c, d).

We then searched 4 microRNA databases and constructed a Venn diagram to identify 144 possible target genes of miR-26a-5p (Fig. 3b), followed by functional enrichment and KEGG analyses using DAVID focusing on ULK1, a target gene of the mTOR signal pathway (Table 1, Additional file 2: Figure S2a). ULK1 was previously reported to be an important initiator of autophagy [7, 9, 14], and we searched STRING to obtain a ULK1 interaction network (Additional file 2: Figure S2b).

**Table 1:** KEGG analysis of the possible target genes of miR-26a-5p

Term	Count	P value	Genes
Insulin resistance	4	0.02807	PPP1R3D, GSK3B, PRKCD, PTEN
mTOR signaling pathway	3	0.047963	ULK1, ULK2, PTEN
Thyroid hormone synthesis	3	0.063367	ATP1A2, PLCB1, ATF2
cGMP-PKG signaling pathway	4	0.068417	PPP3R1, ATP1A2, PLCB1, ATF2

Based on the results, we hypothesized that SNHG6 promotes CRC cell autophagy through regulation of ULK1 via sponging miR-26a-5p. Furthermore, dual-luciferase reporter assays showed that miR-26a-5p binds to ULK1 (Fig. 3c, e). To explore the relationship between SNHG6 and ULK1, we performed qRT-PCR on 31 pairs of CRC tissues and found a positive correlation between SNHG6 and ULK1 (Fig. 3f, g, P < 0.0001, r = 0.737).

### SNHG6 inhibits miR-26a-5p and promotes ULK1-induced autophagy, whereas miR-26a-5p suppresses ULK1-induced autophagy but has no effect on SNHG6

We used qRT-PCR to show that when SNHG6 was knocked down, miR-26a-5p was upregulated but ULK1 decreased; when SNHG6 was overexpressed, miR-26a-5p was downregulated but ULK1 increased (Additional file 2: Figure S2c–e). We also utilized miR-26a-5p mimics to overexpress miR-26a-5p, resulting in no change in SNHG6 but decreases in ULK1; application of miR-26a-5p inhibitors to suppress miR-26a-5p indicated that ULK1 was increased, with no change in SNHG6 (Additional file 2: Figure S2f, h).

**Fig. 4:**
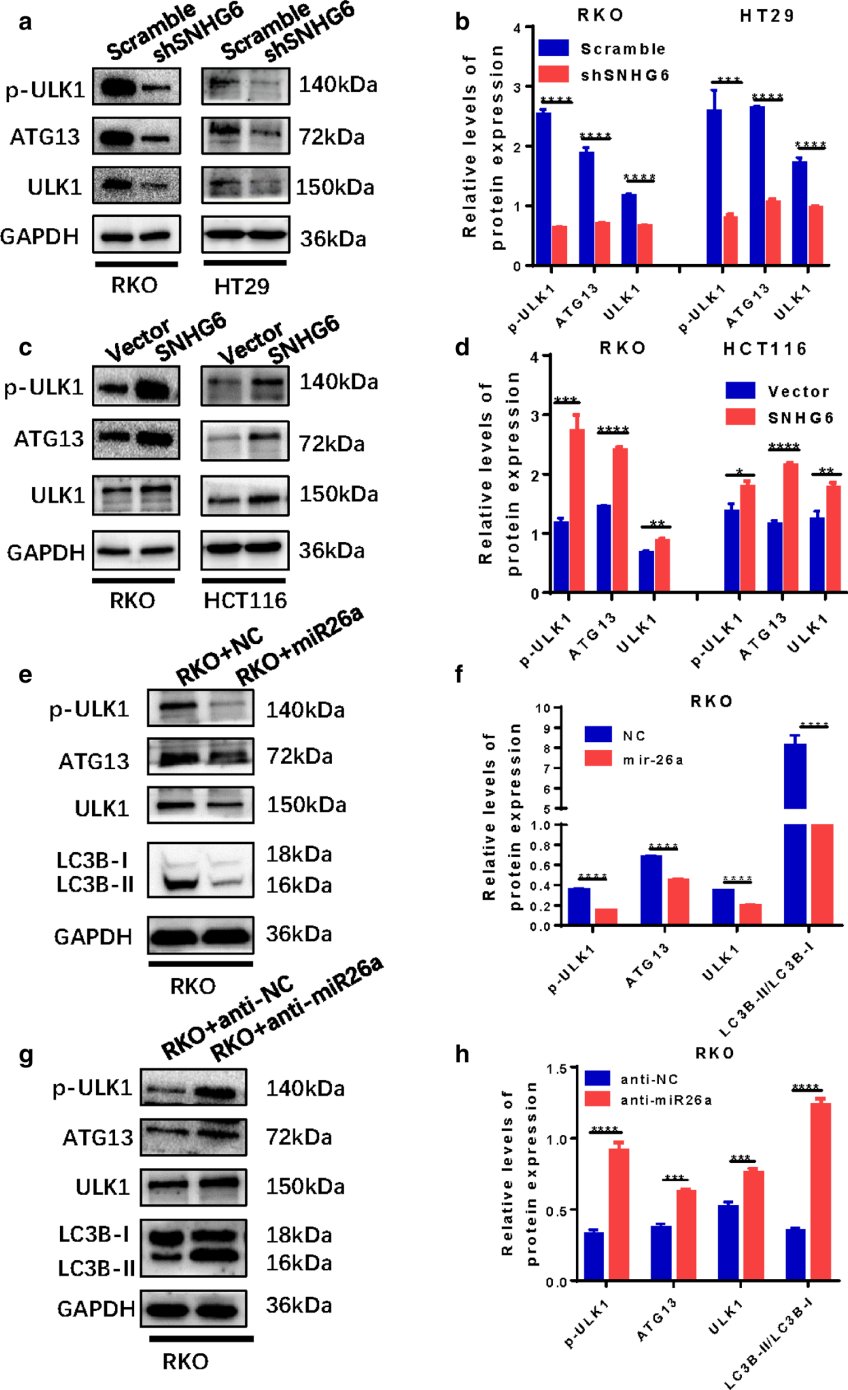
SNHG6 regulates ULK1 via sponging miR-26a-5p. **a**, **b** Knockdown of SNHG6 could decrease the level of p-ULK1, ATG13 and ULK1 proteins, which are parts of autophagy initial unit. **c**, **d** Overexpression of SNHG6 could increase the level of p-ULK1, ATG13 and ULK1 proteins. **e**, **f** Overexpression of miR-26a-5p could decrease autophagy signal pathway proteins. **g**, **h** Knockdown of miR-26a-5p could increase autophagy signal pathway proteins. ns P > 0.05, *P < 0.05, **P < 0.01, ***P < 0.001, ****P < 0.0001, data was shown as the mean ± SD

Next, we used western blotting to evaluate ULK1 and autophagy pathway marker proteins. SNHG6 knockdown in CRC cells resulted in decreases in p-ULK1, ATG13 and ULK1 proteins which are components of the autophagy initiation complex (Fig. 4a, b); in contrast, the levels of these proteins were upregulated when SNHG6 was overexpressed in CRC cells (Fig. 4c, d). Furthermore, we evaluated relationships among miR-26a-5p, ULK1 and autophagy pathway proteins and found the same results when miR-26a-5p was overexpressed as observed with SNHG6 knockdown (Fig. 4e, f). However, when we inhibited miR-26a-5p, we obtained the same results as observed with SNHG6 overexpression (Fig. 4g, h).

### SNHG6 regulates autophagy by targeting ULK1 via sponging miR-26a-5p

**Fig. 5:**
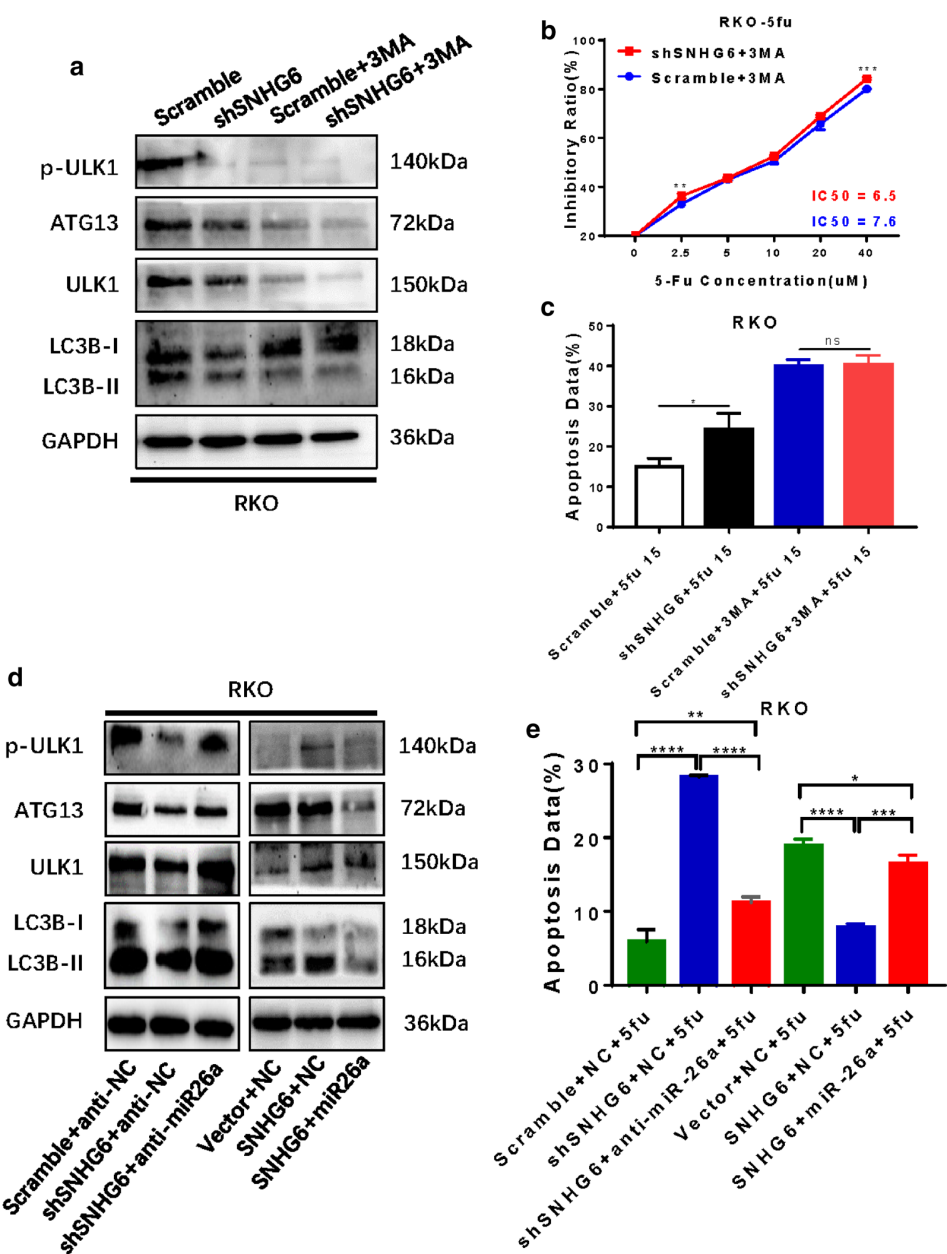
Functional verification experiments confirm SNHG6 could regulate ULK1-induced autophagy via sponging miR-26a-5p. **a**–**c** 3-MA could reduce the difference of autophagy pathway proteins, IC50 and 5FU-induced apoptosis between RKO-shSNHG6 cells and RKO-Scramble cells. **d** miR-26a-5p inhibitors could bring back autophagy pathway proteins of RKO-shSNHG6 cells and overexpression of miR-26a-5p could decrease them in RKO-SNHG6 cells. **e** miR-26a-5p inhibitors could reduce RKO-shSNHG6 cells apoptosis but miR-26a-5p brought it back in RKO-SNHG6 cells. ns P > 0.05, *P < 0.05, **P < 0.01, ***P < 0.001, ****P < 0.0001, data was shown as the mean ± SD

To confirm the function of the SNHG6/miR-26a-5p/ULK1 axis in autophagy in verification tests, we used the autophagy pathway inhibitor 3-methyladenine (3-MA), which has been applied to treat advanced CRC [22]. After 3-MA treatment, the difference in autophagy pathway proteins between RKO-shSNHG6 cells and RKO-Scramble cells was reduced (Fig. 5a). The IC50 of these two groups became more similar (Fig. 5b), and the difference in 5-FU-induced apoptosis was also reduced (Fig. 5c).

Next, we performed recovery experiments. Addition of miR-26a-5p inhibitors restored the mRNA level of ULK1 but had few effects on SNHG6 in RKO-shSNHG6 cells (Additional file 2: Figure S2i–k). Moreover, miR-26a-5p inhibitors also restored levels of autophagy-related proteins, including p-ULK1, ATG13, ULK1 and LC3-II, and decreased RKO cell apoptosis when SNHG6 knockdown increased it. Additionally, overexpression of miR-26a-5p in RKO-SNHG6 cells reduced levels of autophagy-related proteins and promoted RKO cell apoptosis (Fig. 5d, e). These recovery experiments indeed confirmed that SNHG6 regulates autophagy by targeting ULK1 via sponging miR-26a-5p.

## Discussion

Resistance to chemotherapy plays a significant role in CRC mortality [5]. Although precision medicine and targeted therapies offer new hope for treating CRC, chemotherapy remains an important therapy for most CRC patients. However, as 30% to 50% of patients relapse with chemotherapy-resistant disease, there is an essential need to understand the genetic and molecular mechanisms that contribute to chemotherapy resistance [5, 23, 24]. Because cancer development and progression cannot be fully explained by the coding genome, studies on mechanisms leading to therapy resistance have also focused on noncoding RNAs [25]. In our previous study, we found that the lncRNA SNHG6 was significantly upregulated in CRC and promoted proliferation, invasion and migration in colorectal cancer cells [18]. Moreover, SNHG6 is reported to play an oncogenic function in many types of tumors, such as hepatocellular carcinoma [26, 27], gastric cancer [28, 29], CRC [18, 30–32], lung adenocarcinoma [33], and bladder cancer [34]. However, no report has shown that SNHG6 has a role in chemoresistance. Therefore, we sought to determine whether SNHG6 is also involved in this process and found that SNHG6 promotes 5-FU chemoresistance in RKO cells. We also observed lower levels of apoptosis and higher levels of autophagy in our established 5-FU-resistant RKO cells (RKO/5-FU).

Autophagy plays a key role in the maintenance of cellular homeostasis and adaptation to stress in both normal and malignant cells [10, 35]. Each step of autophagy is under the control of specific autophagy complexes, the activity of which is directly or indirectly regulated by stress signaling pathways [8]. Autophagy has opposing, context-dependent roles in cancer, and interventions to both stimulate and inhibit autophagy have been proposed as cancer therapies [8]. Accordingly, we evaluated the relationship between SNHG6 and autophagy because SNHG6 is able to promote autophagy and inhibit RKO cell apoptosis. Thus, we hypothesized that SNHG6 might regulate autophagy to promote drug resistance in CRC cells.

There is a broad range of estimates for the number of lncRNA genes in mammals, from less than 20,000 to over 100,000 in humans [36]. Nevertheless, the function and biological relevance of the vast majority of lncRNAs remain unclear [16]. LncRNAs affect gene regulation through multiple mechanisms. For example, nuclear-retained lncRNAs affect transcription and epigenetic regulation of genes [37, 38], whereas cytoplasmic lncRNAs can regulate the activities of interacting proteins and microRNAs (miRNAs) in a stoichiometric manner [39, 40]. One hypothesis for assigning lncRNA function is the ceRNA hypothesis, which proposes that specific RNAs can impair miRNA activity through sequestration, thereby upregulating miRNA target gene expression [17]. This hypothesis is supported by experimental evidence for an accumulating number of lncRNAs, particularly circRNAs [41–43], pseudogene-derived lncRNAs [44], and other non-coding RNA [45]. In our study, we attempted to ascertain whether SNHG6 also regulates autophagy through the ceRNA network. We searched DIANA tools LncBase Predicted v.2 and LncACTdb 2.0 to find possible microRNAs that may bind to SNHG6. We then performed qRT-PCR and found that among all predicted microRNAs, miR-26a-5p exhibited a change completely opposite to that of SNHG6, indicating miR-26a-5p as a candidate. Our dual-luciferase reporter assays confirmed our hypothesis. MiR-26a-5p has already been reported to bind to SNHG6 in CRC [31], hepatocellular carcinoma [25] and lung adenocarcinoma [32]. However, there are no reports on the mechanism of SNHG6 and autophagy in CRC. Therefore, we used bioinformatics analysis to identify target genes of miR-26a-5p that might regulate autophagy and obtained ULK1.

**Fig. 6:**
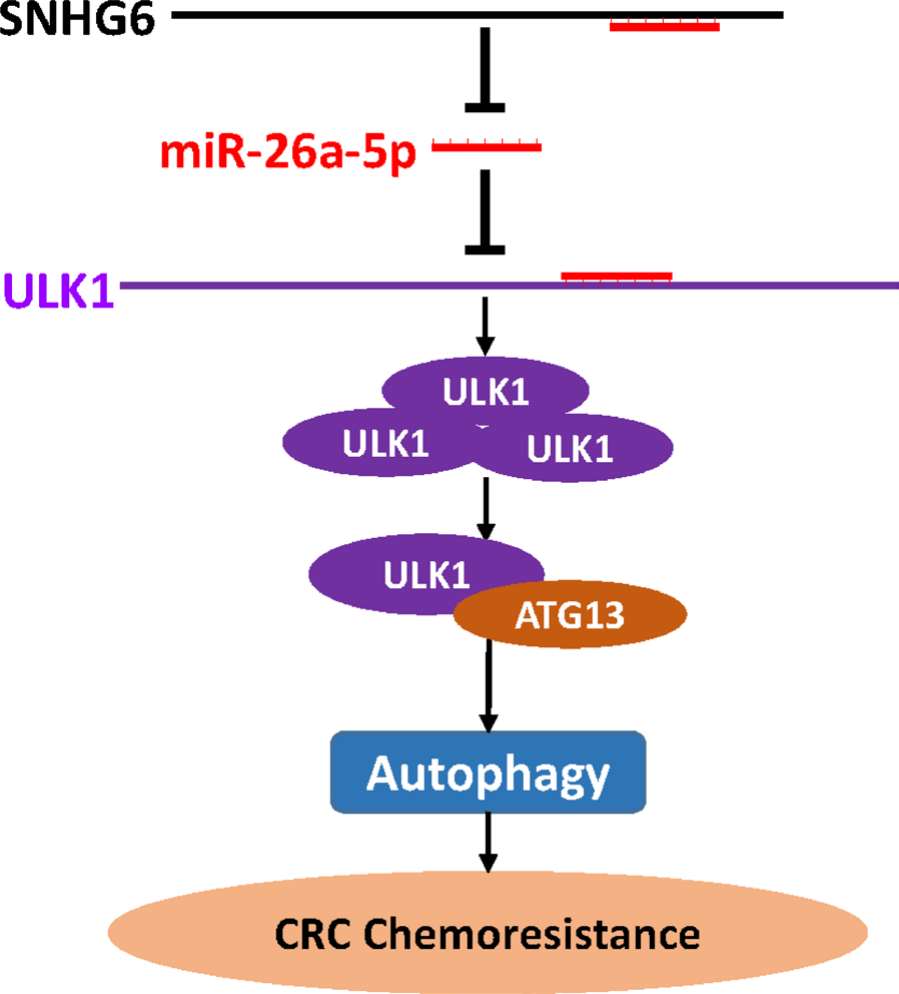
A Schematic model of SNHG6 regulates CRC chemoresistance through ULK1-induced autophagy via sponging miR-26a-5p

ULK1, known as an ortholog of yeast Atg1, is the serine-threonine kinase and the autophagic initiator in mammals [14]. The ULK1 complex is essential for transmitting stress signals to the site where the autophagosome will form, mainly under nutrient- or energy-deprived conditions, by both mediating activating phosphorylation of downstream autophagy proteins and playing non-catalytic, scaffolding roles [12]. Therefore, we focused on ULK1 as participating in SNHG6-mediated regulation of autophagy. We confirmed binding between ULK1 and miR-26a-5p by dual-luciferase reporter assays. Moreover, qRT-PCR and western blot analyses revealed that SNHG6 is able to inhibit miR-26a-5p to regulate ULK1-induced autophagy but that miR-26a-5p does not regulate SNHG6. Moreover, our rescue experiments provide more evidence of the function of the SNHG6/miR-26a-5p/ULK1 axis in autophagy (Fig. 6).

## Conclusions

In summary, our study revealed that SNHG6 enhances chemoresistance through ULK1-induced autophagy via sponging miR-26a-5p (Fig. 6). These findings suggest that SNHG6 is an important therapeutic target for CRC.

## Supplementary information

**Additional file 1: Figure S1.** (a, b) RKO/5-FU cells had lower level of cell apoptosis and higher level of LC3-II. (c) RKO/5-FU cells had higher IC50 than RKO cells. (d) Graphs of cell apoptosis. ns P > 0.05, *P < 0.05, **P < 0.01, *** P < 0.001, ****P < 0.0001, data was shown as the mean ± SD.

**Additional file 2: Figure S2.** (a) KEGG analysis showed ULK1 might be one of the downstream target genes of miR-26a-5p and related to mTOR signal pathway. (b) STRING analysis showed ULK1 interaction network. (c–e) qRT-PCR showed SNHG6 could inhibit miR-26a-5p but upregulate ULK. (f–h) qRT-PCR showed miR-26a-5P could inhibit ULK1 but have no effect on SNHG6 (i-k) qRT-PCR showed inhibiting miR-26a-5p in RKO-shSNHG6 cells could bring back ULK1. ns P > 0.05, *P < 0.05, **P < 0.01, ***P < 0.001, ****P < 0.0001, data was shown as the mean ± SD.

## Data Availability

Not applicable.

## Abbreviations

lncRNA: long noncoding RNA
SNHG6: small nucleolar RNA host gene 6
CRC: colorectal cancer
qRT-PCR: quantitative real-time PCR
ULK1: unc-51-like autophagy activating kinase 1
5-FU: 5-fluorouracil
FIP200: focal adhesion kinase family-interacting protein of 200 kDa
ceRNA: competitive endogenous RNA
miRNA: microRNA
IRB: institutional review board
CCK-8: Cell Counting Kit-8
IC50: half-maximal inhibitory concentration
