# Supplementary figures and images for: LncRNA SNHG6 promotes chemoresistance through ULK1-induced autophagy by sponging miR-26a-5p in colorectal cancer cells
Source: Cancer Cell Int. 2019 Sep 9;19:234. doi: 10.1186/s12935-019-0951-6 (PMC6734319; doi:10.1186/s12935-019-0951-6)

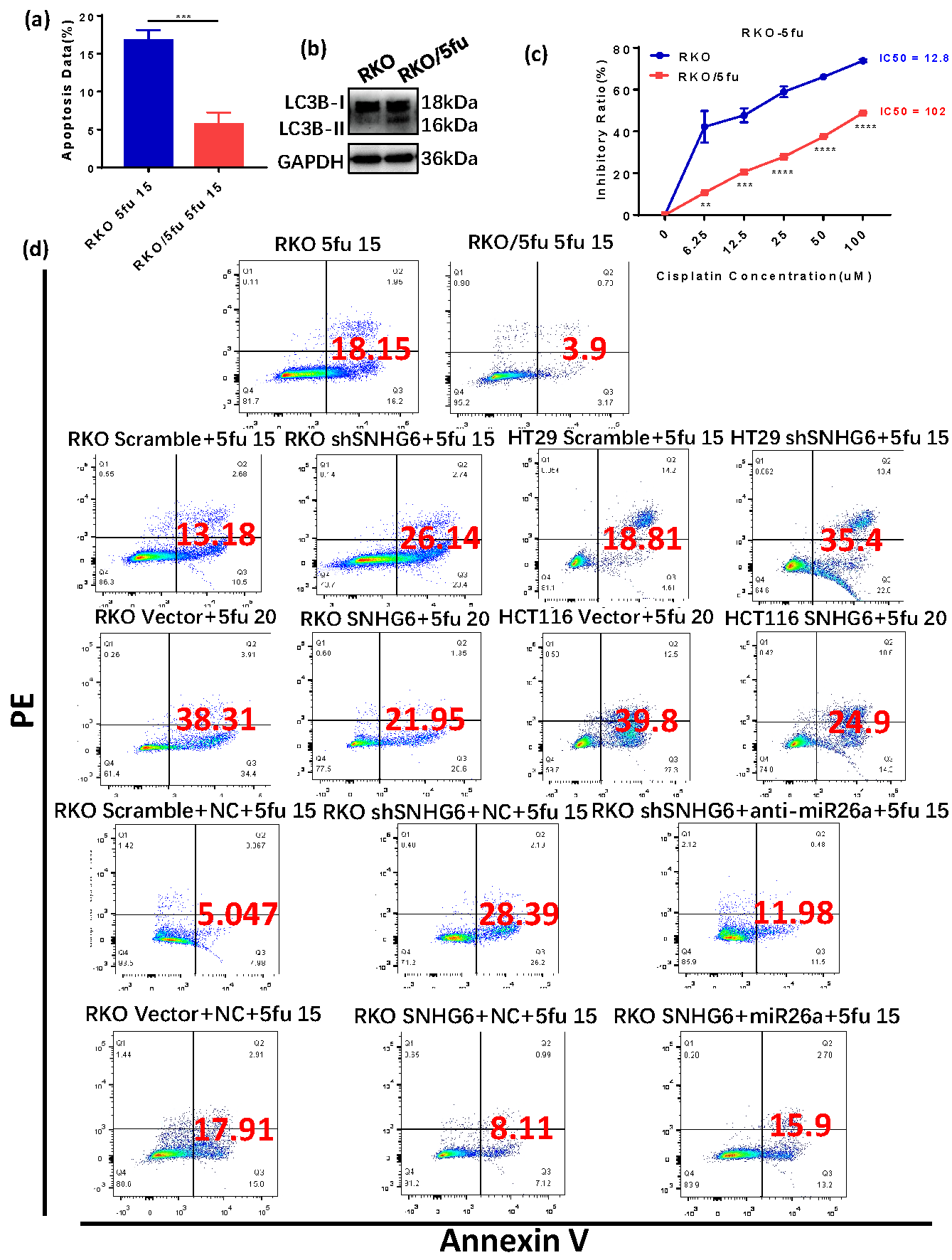

Supplement: Supplementary file 1 — Additional file 1: Figure S1. (a, b) RKO/5-FU cells had lower level of cell apoptosis and higher level of LC3-II. (c) RKO/5-FU cells had higher IC50 than RKO cells. (d) Graphs of cell apoptosis. ns P > 0.05, *P < 0.05, **P < 0.01, *** P < 0.001, ****P < 0.0001, data was shown as the mean ± SD. [file 12935_2019_951_MOESM1_ESM.tiff]

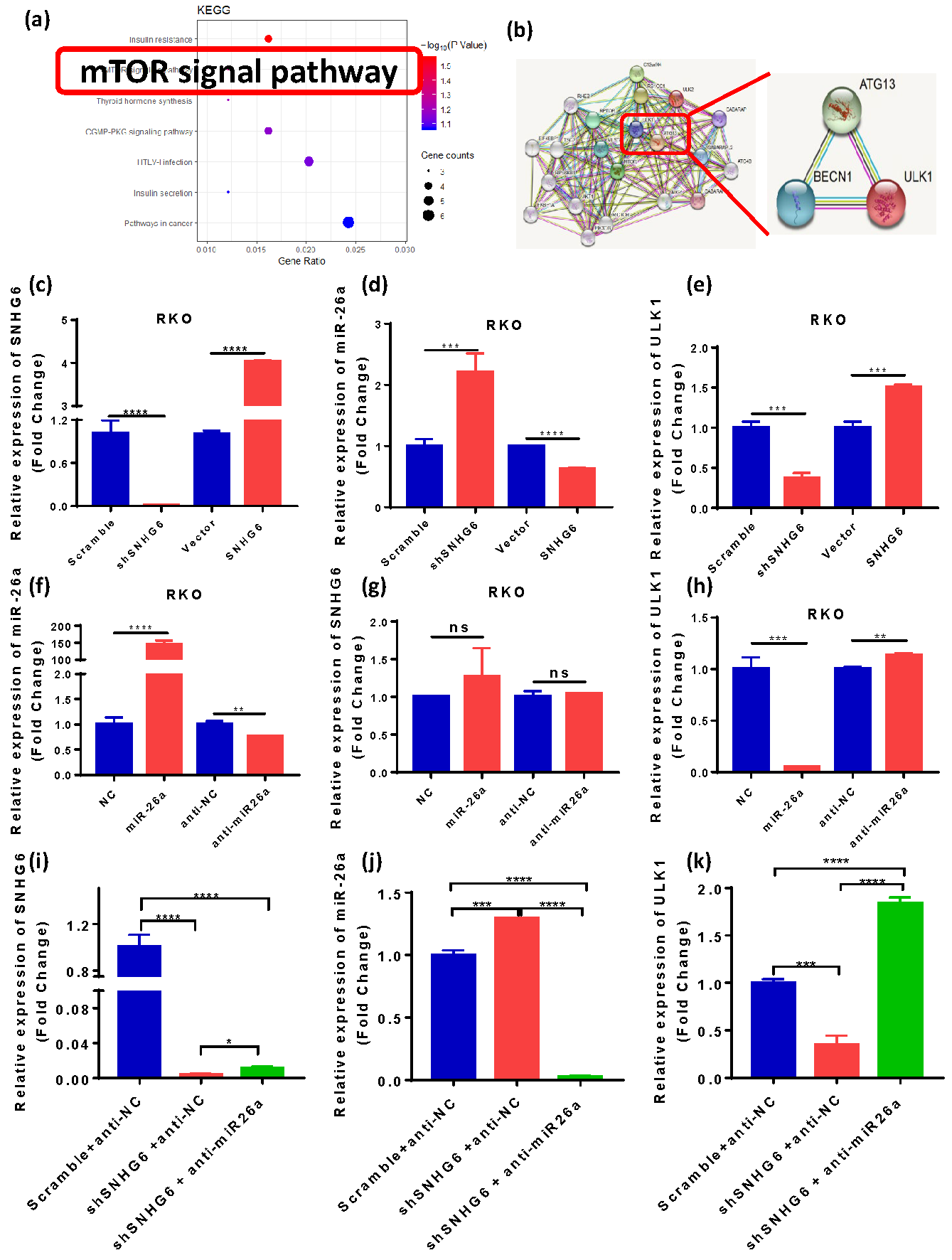

Supplement: Supplementary file 2 — Additional file 2: Figure S2. (a) KEGG analysis showed ULK1 might be one of the downstream target genes of miR-26a-5p and related to mTOR signal pathway. (b) STRING analysis showed ULK1 interaction network. (c–e) qRT-PCR showed SNHG6 could inhibit miR-26a-5p but upregulate ULK. (f–h) qRT-PCR showed miR-26a-5P could inhibit ULK1 but have no effect on SNHG6 (i-k) qRT-PCR showed inhibiting miR-26a-5p in RKO-shSNHG6 cells could bring back ULK1. ns P > 0.05, *P < 0.05, **P < 0.01, ***P < 0.001, ****P < 0.0001, data was shown as the mean ± SD. [file 12935_2019_951_MOESM2_ESM.tiff]
